# An *Ex-vivo* Shear and tensile bond strengths of orthodontic molar tubes bonded using different techniques 

**DOI:** 10.4317/jced.53545

**Published:** 2017-03-01

**Authors:** Elham Abu-Alhaija, Mohammad Jaradat, Ahed Alwahadni

**Affiliations:** 1 Professor, Department of Preventive Dentistry, Faculty of Dentistry, Jordan University of Science and Technology, Irbid-Jordan; 2 Master student, Department of Preventive Dentistry, Faculty of Dentistry, Jordan University of Science and Technology, Irbid-Jordan; 3 Professor, Department of Prosthodontics, Faculty of Dentistry, Jordan University of Science and Technology, Irbid-Jordan

## Abstract

**Background:**

Molar bonding procedures need continuous improvement to be widely accepted clinically and eventually replace molar bands.

**Material and Methods:**

The purpose of this study was to determine the effects of enamel micro-abrasion and silane coating of the base of molar tubes on shear and tensile bond strengths of orthodontic molar tubes. A total of 200 third molars were randomly allocated into five groups of 40 teeth as follows: group 1: molar tubes bonded to etched teeth (37% phosphoric acid gel; control group); group 2: molar tubes bonded to etched teeth (37% phosphoric acid) with the addition of silane to the base of molar tubes; group 3: molar tubes bonded to teeth pre-treated with 18% hydrochloric acid and pumice (micro-abrasion); group 4: molar tubes bonded to teeth pre-treated with microabrasion with the addition of silane to the base of molar tubes; group 5: molar tubes bonded to teeth pre-treated with microabrasion before conventional acid etching combined with the addition of silane to the base of molar tubes. The bond strength testing was performed using a computer control electromechanical universal testing machine.

**Results:**

The highest mean shear and tensile bond strengths were recorded in group 5 (13.81±2.54MPa and 13.97±2.29 MPa, respectively). Micro-abrasion alone (group 3) and the combination of enamel micro-abrasion and the addition of silane (group 4) produced bond strength values comparable to the control.

**Conclusions:**

Enamel surface pre-treatment (micro abrasion) before conventional acid etching combined with the addition of silane to the base of the molar tube produced the highest bond strengths among all tested groups.

** Key words:**Molar, shear strength, tensile strength, orthodontic appliances.

## Introduction

Modern fixed orthodontic appliances include bonding of teeth from second molar to second molar. A bonded attachment must withstand forces generated during orthodontic treatment and those transmitted to the teeth during mastication and occlusion. Inability to resist these forces results in bond failure. Bonding of attachments to molars, rather than banding, is a less frequently adopted practice despite the periodontal advantages it confers over molar bands (1). High bond failure rates have been reported for bondable tubes (2).

It has been recommended that the minimum clinically acceptable bond strength is 5.88 - 7.85 MPa (3). Knoll *et al.* (2) evaluated the maximum shear bond strength (SBS) of brackets bonded to anterior and posterior teeth. They reported a mean value of 11±4.0 MPa for attachments bonded to molar teeth. Similarly, Bishara *et al.* (4) recorded 11.8±4.1 MPa for the tested molar tubes. Most recently, Pinzan-Vercelino *et al.* (5) recorded a mean value of 12.53 MPa for the bonded molar tubes.

A variety of bonding protocols has been suggested to maximize the bond strength of the bonded attachments. These include application of silane agent, (6) application of additional layer of resin to the occlusal surface of tube/tooth interface, (5) overetchning and enamel micro-abrasion (7,8).

Microabrasion has been used for demineralized white spot removal (9) When used as preconditioning agent it was claimed to prepare enamel surfaces in a manner similar to acid etching with the possibility of increasing adhesion (10). Gelgör *et al.* (11) performed microabrasion on artificial white spot lesions and showed that this technique has no detrimental effect on enamel. Baysal and Uysal (8) reported that micro-abrasion of the enamel surface improved bond strength of orthodontic brackets bonded to fluorotic enamel.

## Material and Methods

To date, some studies have evaluated the effect of microabrasion on the SBS of molar tubes bonded to fluorosed enamel,7 but none has studied this on normal teeth. Therefore, the aims of this *ex-vivo* study were to determine the effects of enamel micro-abrasion and silane coating of the base of molar tubes on shear (SBS) and tensile (TBS) bond strengths of orthodontic molar tubes and to report on the mode of failure after debond. The null hypothesis tested was there is no difference in shear and tensile bond strengths of orthodontic molar tubes in the different groups.

-Specimens

A total of 200 freshly extracted human molar teeth (third molars) were collected from patients for varying reasons. Careful examination for the buccal surface of each tooth was carried out to ensure that it was sound and free from cracks, caries or restoration which might affect their resistance to experimental loading. Teeth were stored in 10% thymol solution at room temperature for one week. Then they were mounted vertically in cold curing denture base acrylic resin cylinders (height 20 mm and with an 18 mm diameter) with the crowns exposed. The buccal surface of each tooth was polished with fluoride-free pumice slurry for 15 seconds, rinsed and dried.

-Bonding

Teeth were randomly divided into five groups of 40 teeth each. Each group was tested in shear and tension (20 specimens for each test). Standard edgewise metal lower buccal molar tubes with a base surface area of 15.81 mm2 (mesh base, 3M, Monrovia, California, USA) were bonded to the teeth with a different bonding protocol used for each group. In all groups, an adhesive primer (Transbond XT, 3M Unitek, Monrovia, California, USA) was applied on the etched surface and light cured for 10 seconds. The light-cured composite (Transbond XT, 3M Unitek, Monrovia, California, USA ) was applied on the base of the tube and pressed firmly onto the tooth, and the excess adhesive was removed. The adhesive was light cured (Biolux TM, CFON 1163, BIO-ART, Dental Equipment Ltd, Sao Crlos, Brazil) from the mesial and distal for 20 sec each (total time 40 sec).

-Group 1: Molar tubes bonded to etched teeth (37% phosphoric acid gel; control group)

Teeth were etched using 37% phosphoric acid gel (3M ESPE for 30 seconds), rinsed with water for 15 seconds, and dried with oil-free air for 10 seconds until a frosty white appearance was followed by the application of Transbond XT light cured primer and adhesive.

-Group 2: Molar tubes bonded to etched teeth (37% phosphoric acid) with the addition of silane coupling agent to the base of molar tubes

Teeth were etched using 37% phosphoric acid gel followed by the application of Transbond XT light cured primer and adhesive. A thin coat of silane was added to the base of the molar tube before the application of the primer and the composite adhesive to the base of molar tubes.

-Group 3: Molar tubes bonded to teeth pre-treated with 18% hydrochloric acid and pumice (micro-abrasion)

In this group, teeth was treated with 18% hydrochloric acid mixed with fine pumice powder to obtain a slurry form. Teeth were cleaned slowly with pumice and water by using a rubber cup in a slow speed handpiece. This mixture was applied to the buccal surface of each tooth to be bonded. The slurry was agitated into the tooth surface for 5 seconds and then washed off with an air-water spray. The cycle of microabrasion procedure and washing was repeated 10 times on each tooth. This was followed by the application of Transbond XT light cured primer and adhesive.

-Group 4: Molar tubes bonded to teeth pre-treated with microabrasion with the addition of silane to the base of molar tubes 

Enamel surface was treated with 18% hydrochloric acid and pumice (micro-abrasion) and a thin coat of silane was added to the base of molar tubes followed by the application of Transbond XT light cured primer and adhesive.

-Group 5: Molar tubes bonded to teeth pre-treated with microabrasion before conventional acid etching combined with the addition of silane to the base of molar tube

Enamel surface of teeth was treated with 18% hydrochloric acid and pumice (micro-abrasion) followed by acid etching using 37% phosphoric acid and a thin coat of silane was added to the base of molar tubes followed by the application of Transbond XT light cured primer and adhesive.

After polymerization, the specimens were stored in a distilled water bath at 37ºC for 24 hours. Subsequently they were thermocycled from 5ºC to 55ºC and back to 5ºC 500 times. The dwell time at each temperature level was 30 seconds and the transfer time between baths was 10 seconds. Then the specimens were placed in distilled water at 37ºC for a period of 24 hours before being tested in shear and tension.

-Loading of Test Specimens

Bond strength testing was performed using a computer control electromechanical universal testing machine (WDW 20, JINAN testing Equipment, I E Corporation, Shandong China). The results were recorded via a computer electronically connected to the Instron machine. Baty shadomaster machine (Type R11M, BATY and Co Ltd., Victoria Road, West Sussex, UK) was used in order to calculate the projected bonding surface area for all of the molar tubes.

A 0.021X0.025 inch stainless steel wire was ligated into each bracket slot to minimize deformation of the bracket during debonding.

-SBS test

The specimens (100 specimens/20 per each group) were clamped vertically in the testing machine so that the molar tube base was parallel to the direction of the applied (shear) forces. The debonding force was applied in a gingivo-occlusal direction at a crosshead speed of 1 mm per minute.

-TBS test

Specimens (100 specimens/20 per each group) were clamped horizontally in the testing machine so the applied tensile forces were perpendicular to the molar tube base. The crosshead speed of the testing machine was again 1 mm per minute as in the shear test.

-Mode of Bond Failure

The base of the orthodontic molar tube and the bonding areas of the molar teeth were examined visually by one investigator (M.J) using a magnifier with 88 mm diameter lens and 2.5x magnification (G-777-090, Shenzhen Guanyida Optical Production Corp Ltd, China) to determine the amount of adhesive remained on the tooth surface using the modified Adhesive Remnant Index (ARI) by Bishara *et al.* (12) :

1- The entire composite remained on the tooth with distinct impression of the tube base.

2- More than 90% of the composite remained on the tooth surface.

3- More than 10% but less than 90% of the composite remained on the tooth surface.

4- Less than 10% of the composite remained on the tooth surface.

5- No composite resin remained on the enamel.

Statistical analysis was performed using Statistical Package for Social Science (SPSS) computer software (SPSS 15.0, SPSS Inc., Chicago, USA). The mean and standard deviation (SD) of each group were calculated. Comparison between groups was performed using ANOVA. Bonferroni post hoc multiple comparisons were used. Comparison between the different groups in modes of tube failure was carried out using the Chi square test.

## Results

To test the intra-examiner reliability for ARI scores using kappa test 20 molar tubes were choosen randomly to be examined (two times) by the same investigator (M.J) after a period of one week. Kappa values were above 90%.

Means, standard deviations (SD), standard error of the mean (SE) and 95% confidence interval (CI) for the SBS and TBS of the tested groups are shown in  Table 1. Comparison between the five tested groups is shown in  Table 2.

**Table 1:**
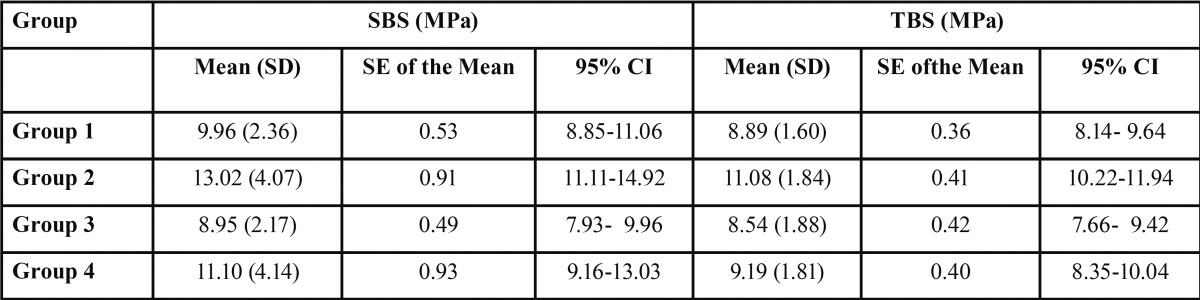
Means, standard deviations (SD), standard errors (SE) of the means and 95 per cent confidence intervals (CI) of SBS and TBS in MegPascals (MPa) for the studied groups.

**Table 2:**
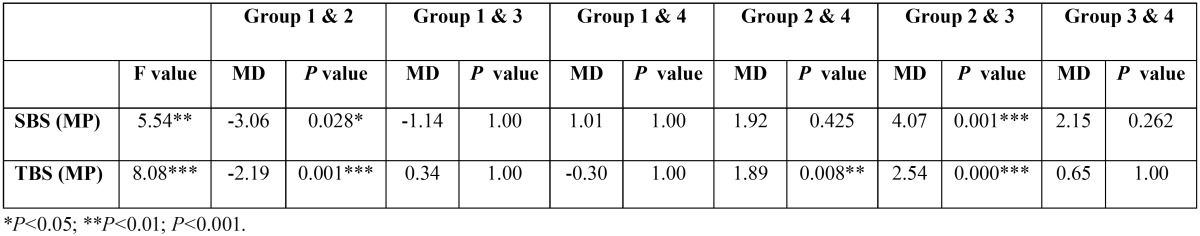
F values, Differences between means (MD) of SBS and TBS and *p* values for the studied groups.

SBS

The highest mean SBS was recorded in group 5 (13.81±2.54 MPa) while the lowest mean SBS was observed in group 3 where the teeth were micro-abraded before bonding (7.73±1.87 MPa).

TBS

The highest mean TBS was recorded in group 5 (13.97±2.29 MPa) while the lowest mean TBS was observed in group 4 (7.38±1.63 MPa).

Compared with the control, SBS and TBS were increased with the addition of silane to the base of molar tubes (*P*=0.037 and *P*=0.008, respectively) and when combined with enamel surface pretreatment with HCL and pumice (micro-abrasion) followed by acid etching (*P*=0.000).

Mode of Bond Failure 

 Table 3 shows the patterns of bond failure for each of the five tested groups (shear and tensile tests).

**Table 3:**
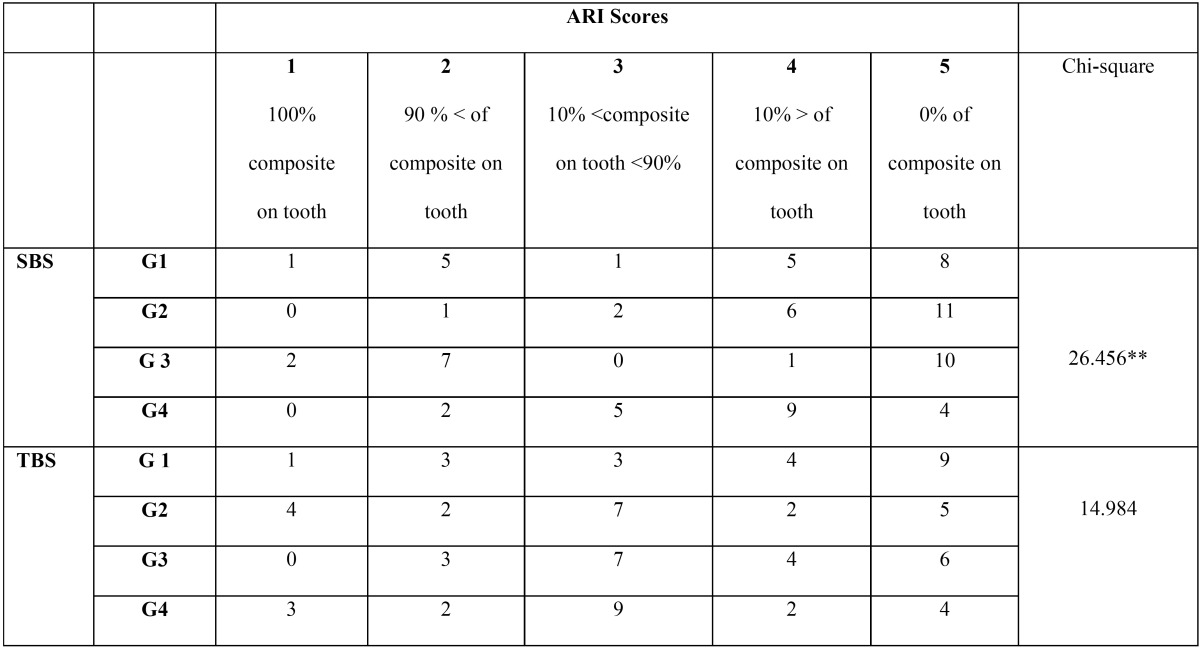
Adhesive Remnant Index (ARI) scores for the tested groups.

SBS

ARI scores revealed that in group 3, failure at adhesive-molar tube interface occurred in 45% of specimens whereas failure at adhesive- enamel surface interface occurred in 70% of specimens in group 4. Differences in ARI scores between studied groups were detected (*P*=0.028).

TBS

ARI scores revealed that in group 1, failure at adhesive-molar tube interface occurred in 65% of specimens whereas failure at adhesive- enamel surface interface occurred in 40% of specimens in group 4. Differences in ARI scores between studied groups were not detected (*P*=0.418).

## Discussion

Several studies have evaluated the failure rate of bonded molars tubes (1,13,14). In their study, Pandis *et al.* (14) reported that the overall failure rate for the molar tubes was 11%. Few studies have tested alternative methods to decrease the failure rate of the bonded molar tubes. Pinzan-Vercelino *et al.* (5) found that the SBS of bonded molar tubes increased when an additional layer of adhesive was applied to the occlusal part of the tooth/tube interface followed by 10 seconds of additional light-curing. Recently, Silva-Benitez *et al.* (7) reported that the use of overetching and the combination of microabrasion and etching improves SBS of flourotic molars.

In the current study, a new protocol for molar tube bonding was tested. The aim was to increase bond strength of molar tubes to withstand the intra oral debonding forces existed during treatment and function. Although many investigators have considered the tensile forces as being less relevant to clinical practice (15), this study aimed also to evaluate the tensile bond strength for each of the tested groups.

In the present study, five groups were tested of 40 specimens each (20 for SBS/ 20 for TBS). Fox *et al.* (16) suggested that at least 20 specimens should be used per test if valid conclusions are to be drawn from *in-vitro* bond strength testing. However, a sample size greater than 10 per group is recommended for bond strength testing of natural teeth where variations in tooth shape exist (15).

Third molars were selected to represent molar teeth because they can be easily collected and they are usually extracted sound and caries free. Also, due to the evolution of more conservative dental treatments, extraction of first and second molars became not frequently done.

Natural teeth were stored in 10% thymol solution at room temperature for one week. Thymol solution has an advantage of having an antifungal action, which is the reason for choosing this substance as a storage media (17). The specimens in the present study were thermocycled from 5ºC to 55ºC and back for 500 cycles in order to simulate the temperature fluctuation that happens inside the oral cavity (18).

Tensile bond strength was measured in addition to SBS to measure the adhesion forces between two surfaces in an attempt to simulate the bond strength when the activations of the appliance are carried out.

In this study, SBS of the control group coincides with the results reported by others (2,4,5,7). The TBS value for the control group was considered adequate for clinical performance (3). Comparison with other studies is not applicable as no previous studies on TBS of molar tubes were reported before.

The SBS for molar tubes when silane was added to the base of molar tubes was higher than that reported for the conventionally bonded orthodontic molar tubes (2,4,5,7). This finding was in agreement with Guan *et al.* (6) who suggested that the combination of sandblasting and silane increased the *in-vitro* bond strength of plastic brackets for orthodontic application. The improved bond strength may be due to the reaction that occurs between tube base and the silanol groups on one side, and the silane molecule co-polymerization that occurs between the silane molecule and the BIS-GMA system (19). Likewise the TBS for orthodontic molar tubes in silane treated molar tubes was higher than other tested groups. Comparison with other studies is not applicable as no previous studies were reported before.

In group 3 where micro-abrasion alone was applied, SBS and TBS were comparable to that of the control. This result coincides with the findings of Royer and Meiers (20) who reported no difference in the shear/peel bond strength when the composite resin was bonded to micro-abraded/phosphoric acid-etched enamel or to enamel etched with phosphoric acid alone. However, when micro-abrasion was combined with the addition of silane to the base of the molar tube, SBS and TBS values were increased.

In group 5, where enamel surface was pre-treated using HCL and pumice (micro abrasion) before conventional acid etching combined with the addition of silane to the base of the molar tube, SBS and TBS values were the highest. This was in agreement with Silva- Benitez *et al.* (7) who suggested that microabrasion followed by acid etching increase SBS of molar tubes bonded to fluorotic molars.

Bond failure in SBS test occurred equally at the tooth-adhesive and tube-adhesive interfaces in the control and microabrasion groups while in TBS test, most of bond failure occurred at tube-adhesive interface (scores 1 and 2). This was in disagreement with Purmal and Sukumaran (21) who reported that 63% of their sample had bond failure at the tube-adhesive interface. Furthermore, Pinzan-Vercelino *et al.* (5) found that most of the bond failure occurred at the enamel/adhesive interface. When silane was added to the base of molar tubes, bond failure occurred predominantly at the enamel-adhesive interface.

The mode of bond failure in the fourth group was at the tooth-adhesive interface which is opposite to what happened in the micro-abrasion group alone. This difference in the mode of bond failure may be due to the addition of the silane to the base of the molar tube which improved the bond strength between the tube base and the adhesive.

When enamel surface was pre-treated using HCL and pumice (micro abrasion) followed by conventional acid etching combined with the addition of silane to the base of the molar tube (group 5), bond failure occurred equally at the tooth-adhesive and tube-adhesive interfaces and majority of bond failure occurred at in the adhesive interface (score 3). This may be explained by the increased bond strength at both enamel adhesive and adhesive tube interfaces by the microabrasion procedure before etching and the silane to the base of molar tube, respectively.

Molar bonding procedures need continuous improvement to be widely accepted clinically and eventually replace molar bands. One limitation of the current study is its *ex-vivo* design. Although *ex-vivo* bond strength studies are useful to provide information about new bonding techniques, their major drawback is the difficulty to simulate the complex nature of the oral environment.

## Conclusions

- Enamel surface pre-treatment (micro abrasion) followed by acid etching combined with the addition of silane to the base of the molar tube produced the highest SBS and TBS among all tested groups.

The null hypothesis was rejected.
